# Colney-Hatch Lunatic Asylum

**Published:** 1852-07-01

**Authors:** 


### COLNEY-HATCH LUNATIC ASYLUM.

The first medical report of the new county asylum for Middlesex (*Colney Hatch*) is now before us. At the date of the report the asylum contained 1004 pauper lunatics, consisting of—males, 370 (exclusive of 12 male children); 613 adult females, and 8 female children. Dr. W. C. Hood, who is the resident physician on the male side, states in his report that 18 of the 411 patients admitted under his care, were discharged cured within the first six months; 1 was relieved, 16 died, and 376 remained in the asylum. The tabular statements appear to have been drawn up with care, and the account of the *post-mortem* examinations are interesting and valuable to the pathologist. Dr. Hood, in his attempt to convey "some idea of the main view taken of each division, and the particular feature characterizing each sub-division" of insanity, observes: "*Mania* must be received in the general acceptation of the term, as comprehending the acute, chronic, and intermittent forms, each combined with the various complications specified." Is this passage not a little obscure? After defining melancholia, Dr. Hood says: "Dementia, imbecility, and idiocy are as familiar as 'household words,' and demand no explanation!" It would have been more prudent for Dr. Hood to have avoided all attempts at definition; but, having entered the field, we think the important divisions of *dementia*, *imbecility*, and *idiocy*, should not have been so summarily dismissed. "Familiar as household words," indeed! We heard a physician associated with a large county asylum not 100 miles from Colney Hatch, define, in a court of justice, "imbecility" to be "*feebleness of mind!*" Dr. Hood should have enlightened us upon this point. Dr. Hood discards altogether from his nosology the term "*melancholia*" and "*monomonia*," and our readers will ask, for what reason? He says, "*the fact of monomania I very much question*;" and why? *because*, "out of 805 cases registered by Dr. Conolly, in his report of the year 1839, *four* are only ascribed to that form." This does not appear, to our humble judgment, to be either satisfactory, conclusive, or logical reasoning. What matters it to us whether Dr. Conolly, or any other doctor, found among 805 lunatics, or 8000—4 or 400 cases of monomania: it would not alter our opinion as to the existence of this form of diseased mind. The discovery of *one* case should settle the question. A man is said, in legal and medical phraseology, to be a monomaniac when he is under the influence of one prominent delusion, and *apparently* sane and rational upon all other points. Does Dr. Hood deny the existence of a large class of insane patients who come within the scope of this definition? To discard the term monomania, and upon such flimsy grounds, appears to us to be very absurd. Dr. Hood may conceive (as others have done before him) that the mind cannot be diseased upon *one* single point, and actually *sane* upon all others; and that to admit this would be to deny the unity of the mind's action. This is a *metaphysical* question which our judges in Westminster-hall, very properly, we think, will not allow medical men to discuss when in the witness-box. Monomania is a form of disease fully recognised by Penil and Esquirol, and all the eminent legal and medical authorities of France, England, and America; and Dr. Hood should have thought twice before he ventured, upon such grounds, to repudiate its existence. Again, Dr. Hood informs us that he has discarded the term *hypochondriasis* from his nosological table, and urges, with great simplicity, as his reason for so doing, that "*it is a state induced by physical derangement, depending principally on the chylopoietic viscera, or uterus, and susceptible of amelioration without calling for the moral treatment or vigilance of a lunatic asylum.*" (p. 39.) If we are to exclude from our vocabulary all the forms of disturbed mind *because* they may be "induced by *physical* derangement," with what class of case shall we fill the wards of our private and public asylums? Surely Dr. Hood is sufficiently conversant with pathological science to know that every form of insanity is "induced by *physical derangement.*" We consider Dr. Hood is not justified in omitting the term hypochondriasis from his nosology, unless he has a more philosophical reason than that assigned in this report for so doing. Has not Dr. Hood inadvertently laid himself open to the charge of *egotism?* When speaking of the treatment of the "*paralytic,*" he observes: "In lieu of soup *I* prefer giving a meat dinner."—"*I* think warm bathing most essential;" and "*I* think the instances where depletion is called for are so rare."—"*I* would fain not acknowledge," &c. We think the personal pronoun is too often and too ostentatiously used in this portion of Dr. Hood's report; thus leaving the impression that we, poor "dogs," are not to enjoy the privilege of "barking," when we are disposed to "open our mouths!" Dr. Hood makes some sensible observations upon the importance of occupation, and has recorded in his report the history of several interesting cases which have been admitted into the asylum. Dr. Davey's report follows. It is well written, and replete with interesting matter. There are no remarks, however, in the report sufficiently novel to justify us in transferring them to our pages. We append, for the perusal of the ratepayers of Middlesex, the creditor and debtor account, having reference to the cost incurred in building this county asylum.

### COST OF CONSTRUCTION OF THE LUNATIC ASYLUM AT COLNEY HATCH.

### DEBTOR.

| | £ | s. | d. | £ | s. | d. |
|---|---|---|---|---|---|---|
| To Amount of Loans raised under authority of General Quarter Sessions | | | | 270,000 | 0 | 0 |
| ,, Cash received for Royalty on Brick Earth . . | 650 | 0 | 0 | | | |
| ,, Cash received for Sale of Trees, &c. . . . . | 139 | 1 | 6 | | | |
| ,, Cash received for sundry Small Rents . . . | 25 | 7 | 0 | | | |
| ,, Profit on Exchequer Bills . . . . . . . . | 2,044 | 10 | 1 | | | |
| | | | | 2,858 | 18 | 7 |
| ,, Amount to be provided . . . . . . . . . . . . . . . . | | | | 16,497 | 6 | 11 |
| | | | | £289,356 | 5 | 6 |

### CREDITOR.

| No. | | £ | s. | d. | £ | s. | d. |
|---|---|---|---|---|---|---|---|
| 1 | By Purchase of Land and Expenses thereon . . . . . . . . | | | | 19,786 | 4 | 8 |
| 2 | ,, Premiums for Designs for Building . . . . . . . . . . | | | | 610 | 8 | 2 |
| 3 | ,, Contract for Building . . . . . . . . . . . . . . . | | | | 138,000 | 0 | 0 |
| 4 | ,, Clock Turret and Clock, Colouring Wards, and painting Chapel Oak . . . . . . . . . . . . . . . . . . . . | | | | 803 | 1 | 0 |
| 5 | ,, Fixtures and Fittings . . . . . . . . . . . . . . . | | | | 18,812 | 6 | 7 |
| 6 | ,, Warming and Ventilating . . . . . . . . . . . . . | | | | 11,583 | 11 | 3 |
| 7 | ,, Hot and Cold Waterworks:— | £ | s. | d. | | | |
| | Pipes, Taps, Baths, &c. . . . . . . . | 4,901 | 6 | 8 | | | |
| | Sinking Well and for Pumps . . . . | 1,294 | 0 | 3 | | | |
| | Steam Engine and Boilers . . . . . | 896 | 14 | 7 | | | |
| | Reservoir . . . . . . . . . . . | 3,179 | 0 | 0 | | | |
| | | | | | 10,271 | 1 | 6 |
| 8 | ,, Gas Buildings, Works and Fittings . . . . . . . . . . | | | | 1,623 | 12 | 10 |
| 9 | ,, Drains . . . . . . . . . . . . . . . . . . . . . . | | | | 4,034 | 13 | 9 |
| 10 | ,, Earthwork, Laying out Grounds, Shrubs, &c. . . . . . . . | | | | 12,281 | 3 | 6 |
| 11 | ,, Formation of Roads, Airing Courts, Ballast, Gravelling and Draining same . . . . . . . . . . . . . . . | | | | 16,430 | 17 | 3 |
| 12 | ,, By Entrance Gates, Lodge, Stabling, and Deadhouse . . . | | | | 1,229 | 16 | 0 |
| 13 | ,, Farm buildings, Slaughterhouses, Dairies, Cottages, &c. . . | | | | 2,000 | 0 | 0 |
| 14 | ,, Chaplain's House, and Fencing thereto . . . . . . . . | | | | 1,322 | 4 | 1 |
| 15 | ,, Railway approach, Railway and Road, Weighing Machine, Engineer's Cottage, and Store Sheds . . . . . . . . . | | | | 1,900 | 0 | 0 |
| 16 | ,, Boundary Walls and Iron Fencing . . . . . . . . . . | | | | 2,527 | 16 | 7 |
| 17 | ,, Furniture, &c.:— | | | | | | |
| | Furniture, Upholstery, &c. . . . . . . | 2,112 | 4 | 1 | | | |
| | Bedsteads and Bedding . . . . . . . | 6,204 | 5 | 8 | | | |
| | Linen Drapery . . . . . . . . . . | 118 | 4 | 9 | | | |
| | Ironmongery, &c. . . . . . . . . . | 1,102 | 1 | 1 | | | |
| | Turnery, &c. . . . . . . . . . . | 419 | 15 | 8 | | | |
| | Earthenware, &c. . . . . . . . . . | 127 | 18 | 11 | | | |
| | | | | | 10,084 | 10 | 2 |
| 18 | ,, Clothing . . . . . . . . . . . . . . . . . . . . . | | | | 2,951 | 16 | 11 |
| 19 | ,, Architect's Commission, Clerks of Works, and Police . . . | | | | 3,448 | 3 | 8 |
| 20 | ,, Incidental Charges:— | | | | | | |
| | Coals, Coke, and Wood . . . . . . . | 822 | 7 | 8 | | | |
| | Printing and Stationery . . . . . | 460 | 4 | 7 | | | |
| | Advertising . . . . . . . . . . | 383 | 19 | 4 | | | |
| | Lithographing Plan of Building . . . | 53 | 5 | 2 | | | |
| | Report on Gas Works and Analysing Water . . . . . . . . . . . | 21 | 0 | 0 | | | |
| | Consecrating Burial Ground, Licensing Chaplain, and for Funeral Furniture, &c. . . . . . . . . . . . . | 53 | 15 | 5 | | | |

| | | | | | | | |
|---|---|---|---|---|---|---|---|
| | Rates and Taxes | 176 | 5 | 7 | | | |
| | Insurance | 62 | 11 | 6 | | | |
| | Salaries and Wages | 1,770 | 1 | 9 | | | |
| | Provisions | 79 | 4 | 1 | | | |
| | Oilman's Stores | 53 | 5 | 3 | | | |
| | Books and Toys | 86 | 15 | 4 | | | |
| | Surgical Instruments, &c. | 68 | 4 | 9 | | | |
| | Marking out Site for Building | 28 | 9 | 0 | | | |
| | Expenses of Laying Foundation Stone | 53 | 10 | 4 | | | |
| | Sundry Petty Disbursements | 343 | 16 | 5 | | | |
| | | | | | 4,516 | 16 | 2 |
| 21 | „ Farm Stock:— | | | | | | |
| | Live Stock | 282 | 4 | 6 | | | |
| | Dead Stock | 144 | 17 | 1 | | | |
| | | | | | 421 | 1 | 7 |
| 23 | „ Law Charges | | | | 1,206 | 6 | 2 |
| | | | | | 265,851 | 11 | 10 |
| 22 & 24 | Liabilities, as per Statement | | | | 23,504 | 13 | 8 |
| | | | | | £289,356 | 5 | 6 |

N.B. These numbers, from 1 to 24, refer to a detailed Statement left with the Clerk of the Peace.

---

